# Prevalence of intestinal parasites in children and domestic animals from two peri-urban neighborhoods in northeastern Argentina

**DOI:** 10.17843/rpmesp.2023.404.12984

**Published:** 2023-12-18

**Authors:** Rumesilda E. Alegre, María de los Ángeles Gómez-Muñoz, Esteban J. Flores-Lacsi, María del Rosario Robles, Francisca Milano

**Affiliations:** 1 Laboratory of Parasite Biology, BioVyP Research Group. Faculty of Exact and Natural Sciences and Surveying., Universidad Nacional del Nordeste, Corrientes, Argentina. Universidad Nacional del Nordeste Laboratory of Parasite Biology, BioVyP Research Group Faculty of Exact and Natural Sciences and Surveying Universidad Nacional del Nordeste Corrientes Argentina; 2 Center for Parasitological and Vector Studies (CEPAVE-CONICET-UNLP), La Plata, Buenos Aires, Argentina. Center for Parasitological and Vector Studies (CEPAVE-CONICET-UNLP) La Plata Buenos Aires Argentina

**Keywords:** Child, domestic animals, parasitic intestinal diseases, prevalence, zoonoses, Argentina

## Abstract

This study aimed to examine the prevalence of intestinal parasites in children and domestic animals from two peri-urban neighborhoods in Corrientes, Argentina. We also evaluated the characteristics of humans, socio-environmental features, and hygiene practices associated with the presence of parasites. Fecal samples were examined using techniques of concentration by sedimentation and flotation. The Graham method was used to diagnose Enterobius vermicularis eggs in children. We carried out the univariate and bivariate analysis of the data. We analyzed 58 dwellings, from which we obtained 146 stool samples from children and 101 from animals. We found at least one parasite species in 54 dwellings (93.1%). We found that 52.7% of children had parasites, mainly *Blastocystis* spp. (35.6%) and *Giardia* spp. (21.2%). We found that 67.32% of the animals had parasites, the most prevalent species being hookworms (60.7%). In conclusion, it is evident that the domestic environment can favor the transmission of these parasites.

## INTRODUCTION

Endoparasites of humans and domestic animals can multiply under favorable conditions, and can be transmitted among the inhabitants of the house ^1^^,^^2^. These favorable conditions are commonly associated to factors related to the individual (age, sex, nutritional status, etc.) ^3^, and/or socio-environmental risk factors such as the inadequate disposition of stool, unsuitable personal hygiene habits, and unequal access to education and health, among others ^4^.

In this regard, the number of slums has increased in Argentina, which lack basic services such as drinking water, electricity and adequate sanitation ^5^. The overall prevalence rates of endoparasites in Argentinean children and their pets have been reported to be 64.8% and 85.7% and 8.9% and 41%, respectively ^6^^,^^7^^,^^8^.

Specifically, 3.6% of dwellings in the province of Corrientes are not adequate for living, 6.5% lack basic sanitary conditions, 11.0% are critically overcrowded, and 37% are located in vulnerable areas (near garbage dumps or flood-prone land) ^9^. However, few parasitological studies have taken these variables into account ^10^.

Therefore, we consider that the city of Corrientes, in northeastern Argentina, could represent a high-risk area for the transmission and maintenance of potentially zoonotic parasites. We also identified important gaps in parasitology research involving human, animal and environmental factors, i.e. operating under the One Health approach, which hinders the interpretation of all these factors together, as well as the opportunity to propose control and prevention measures.

This study aimed to examine the prevalence of intestinal parasites in children and domestic animals in two periurban neighborhoods of the Capital Department (Corrientes, Argentina). In addition, some human characteristics (sex and age), socio-environmental traits and hygiene practices were evaluated as possible risk factors for parasitic infection.

**KEY MESSAGES**

Motivation for the study: There are few reports on intestinal parasites in children and domestic animals in urban areas in Argentina who live in homes with characteristics that favor the maintenance and transmission of parasites of zoonotic importance.

Main findings: More than 50% of children and pets were parasitized, most of them with zoonotic pathogens.

Implications: Our results showed the urgent need to improve sanitary control of children and animals, and to implement activities for the prevention of intestinal parasitosis in the homes analyzed.

## THE STUDY

### Study design

We conducted a cross-sectional, observational, descriptive study. The population was contacted between 2018 and 2021 in an elementary school. The sample size was not calculated; we included all households whose inhabitants agreed to participate. The unit of analysis was the dwelling ^2^.

### Study area

**Figure 1:**
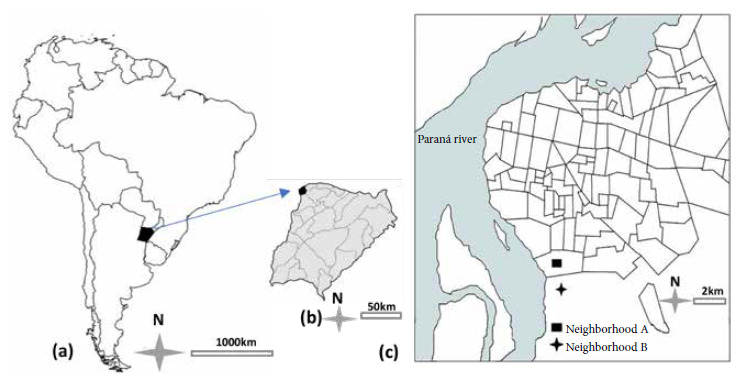
Geographical location of the Province of Corrientes in northeastern Argentina (a), Capital Department (b) and the peri-urban neighborhoods analyzed (c).

The study was conducted in two suburban neighborhoods (A and B) of the Capital Department of Corrientes Province (27°28'00" S, 58°50'00" W), northeastern Argentina (Figure 1). These neighborhoods are characterized by poor sanitary conditions and a high degree of contact with domestic and synanthropic animals, among others.

### Sample collection

A collection kit for each child and pet were delivered to every home. The kit consisted of stool collection bottles with 10% formaldehyde and Graham’s test for the diagnosis of intestinal parasites. Verbal explanations were provided to the participants, which were supplemented by illustrated instructions included in the kit. Children of both sexes aged 0 to 14 years participated in the study. Participants were divided into two age groups: out-of-school children (aged 3 years or less) and school children (aged 4 years or more). Stool and perianal mucosa samples were collected by parents for 5-6 consecutive days. Stool samples from the animals were collected by their owners for 3-4 consecutive days or by the research team. Owners were advised to collect fecal samples from the animals immediately after the animals defecate and in a central area to avoid possible contamination.

### Socio-environmental and demographic data collection

Data were collected through direct observation of the family environment and the application of questionnaires. Data were related to different risk variables or exposure factors, including hygiene practices (Tables 1 and 2).

**Table 1:** Socio-environmental characteristics of the population in the peri-urban neighborhoods analyzed in the city of Corrientes.

Characteristics	n (%) ^a^
Overcrowding (persons per room)	
Yes	28 (50)
No	28 (50)
Presence of domestic animals	
Yes	52 (92.9)
No	4 (7.1)
Floor type (inside the house)	
Cement or other	49 (87.5)
Sand/earth	7 (12.5)
Floor type (outside the house)	
Cement or other	4 (7.1)
Sand/earth	52 (92.9)
Main water source	
Well	17 (30.3)
Community water network (potable)	39 (69.7)
Disposal of human feces	
Latrine	34 (60.8)
Installed bathroom	22 (39.2)
Flood risk	
Never	37 (66.1)
Occasionally	19 (33.9)
Disposal of animal feces	
Stays on the ground	21 (37.5)
Disposed of in bags	35 (62.5)
Wastewater disposal	
Cesspool	55 (98.2)
Outdoor	1 (1.8)
Solid waste disposal	
Collected by the municipality	29 (51.8)
Open air or incineration	27 (48.2)
Disposal of diapers	
Collected by the municipality	23 (41.1)
Open air or incineration	23 (41.1)
Do not use diapers	10 (17.8)
Presence of rodents	
Yes	6 (10.8)
No	46 (82.1)
No data	4 (7.1)
Antiparasitic drug treatment in humans	
Yes	15 (26.8)
No	41 (73.2)
Pharmacological antiparasitic treatment in animals	
Yes	3 (5.3)
No	53 (94.7)
Mother’s education	
Non-schooled	1 (1.8)
Complete primary school	31 (55.3)
Incomplete primary school	7 (12.5)
Complete secondary school	15 (26.8)
Incomplete secondary school	1 (1.8)
No data	1 (1.8)
Father’s education	
Non-schooled	1 (1.8)
Complete primary school	25 (44.6)
Incomplete primary school	6 (10.8)
Complete secondary school	9 (16.0)
No data	15 (26.8)

a Frequency was estimated in relation to the total number of homes with socio-environmental data (N=56).

**Table 2:** Hygiene practices of the child population from peri-urban neighborhoods of the city of Corrientes.

Hygiene practices	n (%)^a^
Frequency of bathing	
More than once a week	20 (13.7)
Every day	126 (86.3)
Hand washing before eating and after using the restroom	
Never	3 (2.0)
Occasionally	52 (35.7)
Always	87 (59.5)
No data	4 (2.8)
Nail maintenance	
Short	119 (81.6)
Long	24 (16.4)
No data	3 (2.0)
Walk barefoot	
Never	28 (19.1)
Occasionally	45 (30.9)
Always	68 (46.6)
No data	5 (3.4)
Play on the ground	
Yes	75 (51.3)
No	67 (45.9)
No data	4 (2.8)

a Frequency was estimated in relation to the total number of children with data on hygiene practices (n=146).

### Parasitological analysis

All fecal samples were examined at the Parasite Biology Laboratory of the Facultad de Ciencias Exactas y Naturales y Agrimensura of the Universidad Nacional del Nordeste (FaCENA-UNNE) in Corrientes, Argentina, using sedimentation and flotation concentration techniques ^11^. Graham’s method was used to evaluate the presence of *Enterobius vermicularis* eggs in children. The identification of the parasitic elements (eggs, larvae, cysts, oocysts) was based on their morphological characteristics and measurements. All families received the results of the parasitological diagnosis of the children in writing. A technical report with the results obtained from children and animals was also provided to the corresponding health authorities.

### Statistical analysis

*Univariate analysis*

Frequencies and percentages were described by univariate analysis. Overall prevalence was calculated as the number of positive analysis units and parasitized hosts positive for at least one species divided by the total number of evaluated houses and analyzed hosts, expressed as percentages; specific richness was defined as the number of parasite species. We also calculated the percentage of monoparasitized and polyparasitized (two or more parasite species per host) participants.

*Bivariate analysis*

Fisher’s Exact Test was used to compare the two neighborhoods and evaluate the association between species pairs, as well as the relationship between age group, sex and parasitosis. Data analysis was performed in the R program (R Core Team 2022) ^12^.

The relationships between the age group of the children and the species found, as well as between sex and species were first evaluated by Multiple Correspondence Analysis. This allowed an overview of the relationships between the variables to then verify such associations (by means of a Chi-square test or Fisher’s exact test). The same method was used to analyze the relationship of socio-environmental characteristics with parasitosis and parasitic species and the relationship of hygiene practices with parasitosis and parasitic species. We did not perform multivariate analysis.

### Ethical aspects

The study was approved by the Secretariat of Science and Technology of the Universidad Nacional del Nordeste (UNNE), Argentina. This research was conducted in accordance with Argentine laws. Sample collection was performed under official permissions in accordance with the Universal Declaration of Human Rights of 1948, the ethical standards established by the Nuremberg Code of 1947, the Declaration of Helsinki of 1964 and successive modifications, as well as the provisions of the National Law 25.326 on personal data protection.

## FINDINGS

### Dwellings

A total of 65 households with 164 children and 205 pets were recorded. At least 58 households (considered for analysis) provided a human and/or animal stool sample. We collected 146 samples from children and 101 from animals, representing a return rate of 89.0% and 49.2%, respectively.

At least one species of parasite and/or non-pathogenic organism from children and/or animal hosts was found in 54 dwellings (93.1%). The specific richness was 17 species, with a maximum of seven species in a single dwelling. The most prevalent protozoa were *Blastocystis* spp. and *Giardia* spp. in 60.3% and 43.1% of dwellings, respectively, while the most prevalent helminths were ancylostoma (46.5%) and *Enterobius vermicularis* (15.5%). A higher overall prevalence was found in neighborhood A (n=30) than in neighborhood B (n=28) (96.6% vs. 89.2%), but this difference was not statistically significant (p > 0.05).

### Socio-environmental analysis

Socio-environmental data were obtained from 56 dwellings, in addition to data on personal hygiene practices of 146 children (Tables 1 and 2). We found statistical associations between solid waste disposal and the presence of *Hymenolepis nana* (p=0.024), as well as between the frequency with which participants bathed and the presence of *Entamoeba coli* (p=0.042) (Table 3).

**Table 3:** Variables selected by statistically significant association ^a^

Variables	Parasitosis	Parasitic species ^b^	
		*Hymenolepis nana*	*Entamoeba coli*
Age group			
School-age children	p=0.048	-	-
Non-school-age children	Reference	-	-
Solid waste disposal			
Collected by the municipality	-	p=0.024	-
Open air or incineration	-	Reference	-
Frequency of bathing			
More than once a week	-	-	Reference
Every day	-	-	p=0.042

a Fisher’s exact test was used to evaluate the independence of the variables.

b The other parasitic species were evaluated, but only those with a significant association with the variables are shown.

### Parasitological analysis in children

Seventy-two girls and 74 boys aged 0 to 14 years (45 not attending school and 101 attending school) were analyzed. At least one parasite species was found in 77 samples (52.7%). The specific richness was six species, with the protozoan *Blastocystis* spp. (35.6%) being the most prevalent species, followed by *Giardia* spp. (21.2%). We found a higher presence of parasites in children of school age (4 years and older) than in those who were not of school age (3 years and younger) (40.4% and 12.3% respectively; p< 0.05) (Table 4).

Most samples had a single parasite species, on the other hand, samples with multiple parasites had a maximum of four species. A statistically significant association was found between the presence of *Giardia* spp. and *E. vermicularis* (x2=7.3; p< 0.05).

### Parasitological analysis in animals

A total of 101 fecal samples from domestic animals were analyzed; 68 (67.32%) had at least one parasite species. The specific richness was 15 species. Most of the animals analyzed presented only one parasite species (Table 4).

**Table 4:** General and parasite species prevalence in children (N=146) and animals (N=101) from two peri-urban neighborhoods of the Capital Department, Province of Corrientes.

Groups or parasitic species	Number of positive samples in children n (%)	Age group Number of positive samples n (%)		Number of positive samples in animals n (%)				
		School-age children	Non-school-age children	Dogs (n=84)	Horses (n=8)	Pigs (n=5)	Cats (n=2)	Rabbits (n=2)
Overall	77 (52.7)	18 (12.3)	59 (40.4)	60 (71.4)	2 (25.0)	4 (80.0)	1 (50.0)	1 (50.0)
Polyparasitized	28 (36.3)	6 (33.3)	23 (38.9)	16 (26.6)	0	1 (25.0)	1 (100)	0
Monoparasitized	49 (63.6)	12 (66.6)	36 (61.0)	44 (73.3)	2 (100)	3 (75.0)	0	1 (100)
Parasites								
Protozoa	70 (47.9)	17 (11.6)	55 (37.6)	18 (21.4)	2 (25.0)	3 (60.0)	1 (50.0)	1 (50.0)
*Blastocystis* spp.	52 (35.6)	13 (8.9)	39 (26.7)	2 (3.5)	0	0	0	1 (50.0)
*Coccidia*	0	0	0	2 (2.3)	2 (25.0)	1 (20.0)	0	0
*Eimeria* spp.	0	0	0	1 (1.1)	0	0	0	0
*Entamoeba coli*	10 (6.8)	2 (1.3)	8 (5.4)	1 (1.1)	0	1 (20.0)	0	0
*Giardia* spp.	31 (21.2)	9 (6.1)	22 (15.0)	11 (13.0)	0	0	0	0
*Iodamoeba bustschlii*	0	0	0	0	0	1 (20.0)	0	0
*Isospora canis*	0	0	0	1 (1.1)	0	0	1 (50.0)	0
Helminths	24 (16.4)	4 (2.7)	18 (12.3)	53 (63.0)	0	2 (40.0)	1 (50.0)	0
*Ascaris lumbricoides*	3 (2.0)	1 (0.6)	2 (1.3)	0	0	0	0	0
*Dipylidium caninum*	0	0	0	2 (2.3)	0	0	0	0
*Enterobius vermicularis*	15 (10.2)	2 (1.3)	12 (8.2)	0	0	0	0	0
Ancylostoma	0	0	0	51 (60.7)	0	0	1 (50.0)	0
*Hymenolepis nana*	5 (3.4)	1 (1.3)	4 (2.7)	1 (1.1)	0	0	0	0
*Oesophagostomum* spp.	0	0	0	0	0	2 (40.0)	0	0
*Spirometra* spp.	0	0	0	2 (2.3)	0	0	0	0
*Strongyloides* larvae	0	0	0	1 (1.1)	0	0	0	0
*Toxocara canis*	0	0	0	7 (8.3)	0	0	1 (50.0)	0
*Trichiuris vulpis*	0	0	0	4 (4.7)	0	0	0	0

## DISCUSSION

Infected animals eliminate parasitic stages through their feces and contaminate the environment ^13^. In this sense, our results show that 58.6% of the dwellings were contaminated with parasitic species (particularly geohelminths) found in animal feces, most of them with zoonotic potential. We found that 39.6% of the households were contaminated, in addition to the presence of parasitic species in children’s feces; 32.7% of the dwellings recorded only the presence of parasitic species in children’s feces (mainly zoonotic protozoa). Therefore, it is evident that in these dwellings the conditions for the development of the life cycle, maintenance and transmission of both helminths and protozoa were optimal and constitute a serious epidemiological scenario for public health.

Parasites were found in more than 50% of the children, most of them presented only one species. This coincides with previous research carried out in Corrientes ^10^^,^^14^ and other areas of Argentina ^2^^,^^15^. The most prevalent protozoan species were *Blastocystis* and *Giardia* (35.6% and 21.2%, respectively), while *Entamoeba coli* was the least prevalent (6.8%). This pattern has been reported in infant populations in Argentina ^7^ and in the province of Corrientes, with prevalence rates similar to our results ^10^; however, more recently, locally, the prevalence of *Blastocystis* has been found to be considerably lower (16.7%) ^14^.

Worldwide, *Giardia* is known to be one of the main parasites causing diarrheal diseases (not viral or bacterial) in humans and other mammals ^16^; while *Blastocystis* has been associated with different intestinal and extraintestinal diseases in humans ^17^. *Entamoeba coli* is commensal, but is an indicator of environmental fecal contamination. These three protozoa share the same fecal-oral transmission pathway and their presence is associated with contaminated water or consumption of raw vegetables with feces from infected hosts ^18^. Deficient personal hygiene habits, particularly hand washing, have also been described ^19^. In that sense, it has been reported that parents tend to over-report their children’s hand washing behaviors, which would systematically diminish any apparent benefit and in some cases may even show negative effects ^20^. In this sense, although mothers stated that most children washed their hands before eating and after leaving the bathroom, we found deficiencies in children’s general hygiene, a situation also reported by school authorities. Therefore, this type of result should be validated with a methodology that allows direct observation. Surprisingly, the presence of *E. coli* was associated with the frequency with which individuals bathed, but 86.3% of the mothers stated that their children bathed every day, supporting the need for direct observation.

As for animals, *Giardia* and *Blastocystis* were found in fecal samples from dogs (13.0% and 3.5%, respectively) and in one rabbit. *Entamoeba coli* was found in one dog and one pig. In the city of La Plata, Cociancic *et al*. ^2^^)^ reported a similar prevalence rate in dogs for *Giardia* and a higher prevalence for *E. coli* (10.3%), but they did not find *Blastocystis*. The prevalence was even lower for these protozoa in dogs from another locality in Argentina ^15^. In short, despite the low prevalence of these parasites in pets, we establish the role of animals as a source of environmental contamination and disseminators or as possible foci of animal-animal or human-animal transmission, in which case molecular studies are necessary to verify possible cross-transmissions.

As for helminths, we mainly found *Enterobius vermicularis* (10.2%) and, to a lesser extent, *Hymenolepis nana* (3.4%) and *Ascaris lumbricoides* (2.0%). *Enterobius vermicularis* has been widely reported in all provinces of Argentina, with a prevalence rate ranging from 13.6% to 50.9% ^14^. Different studies associated its presence with onychophagia ^2^, poor nail and hand washing, overcrowding, and sharing beds and clothes ^21^. In our study, it is possible that the low prevalence is related to inefficient parental sampling.

*Hymenolepis nana* commonly infects humans and rodents. A prevalence rate similar to that found by our study has been reported in Argentina ^(^^14^^,^^22^. In contrast, a prevalence rate higher than 20.0% was reported in the province of Misiones ^7^. Our results show that the presence of this parasite was associated with solid waste disposal. However, adults stated that garbage is collected in bags and then removed by the municipal collection service in all households where this helminth was found. It is important to note that we observed excessive accumulation of garbage both at the household level and in specific sectors of the neighborhoods evaluated. Therefore, there is evidence of inadequate waste disposal in this community, and we consider that this represents a risk, given the presence not only of rodents but also of domestic animals that were in direct contact with the waste.

*A. lumbricoides* was found in three children in three different dwellings. In contrast, a high prevalence of this parasite (38.9%) has been reported in other areas of Argentina ^23^. Its presence in the environment is mainly related to open defecation, climatic conditions and soil, the latter determining the viability and maturation of the eggs. In Brazil, Gonçalves *et al*. ^24^ determined the absence of latrines as a risk factor, showing that in South America, indigenous people usually live in conditions of extreme poverty.

Regarding the animals, ancylostoma, *Toxocara canis* and *Trichuris vulpis* were reported to be most prevalent in dogs (60.7%, 8.3% and 4.7%, respectively). This demonstrates high canine fecal contamination and poor pet health care in households. Similar results were reported in the city of Corrientes by Milano *et al*. ^1^, and higher values were reported in the city of La Plata by Cociancic *et al*. ^3^. *Ancylostoma* larvae and *T. canis* can be transmitted to humans and cause cutaneous larva migrans syndrome and neural larva migrans syndrome, respectively ^25^. *Trichuris vulpis* is a nematode of the large intestine of dogs and is of importance in veterinary medicine.

On the other hand, in agreement with Rivero *et al*. ^15^, co-infection of *Giardia* spp. and *E. vermicularis* was the most common and statistically significant, this could be due to the fecal-oral route of transmission that both parasite species share.

Regarding demographic variables, we found a significant association between age group and parasitosis, with school children being the group with the highest prevalence of parasites. Similar results have been reported by Rivero *et al.*^15^^)^ and Navone *et al.*^14^ This finding could be attributed to the fact that schoolchildren maintain close contact with the foci of parasitic infection through play and insufficient hygiene habits ^26^.

The sample size was one of the limitations of our study, because we worked with a low number of dwellings and a low number of animal and human feces.

In conclusion, we found that a large number of the homes were contaminated with parasitic species, mostly helminths and protozoa of zoonotic importance, which shows the urgent need to improve sanitary control of children and animals in the area.
