# Supplementary material for: Prevalence of intestinal parasites in children and domestic animals from two peri-urban neighborhoods in northeastern Argentina
Source: Rev Peru Med Exp Salud Publica. 2023 Dec 18;40(4):466–73. doi: 10.17843/rpmesp.2023.404.12984 (PMC11138830; doi:10.17843/rpmesp.2023.404.12984)
Supplement: Translation

# Translation: Prevalencia de parásitos intestinales en niños y animales domésticos en dos barrios periurbanos del nordeste de Argentina

**Authors:** Rumesilda E. Alegre, María de los Ángeles Gómez-Muñoz, Esteban J. Flores-Lacsi, María del Rosario Robles, Francisca Milano

**Affiliations:** 1 Laboratorio de Biología de los Parásitos, Grupo de Investigación BioVyP. Facultad de Ciencias Exactas y Naturales y Agrimensura, Universidad Nacional del Nordeste, Corrientes, Argentina.; 2 Centro de Estudios Parasitológicos y de Vectores (CEPAVE-CONICET-UNLP), La Plata, Buenos Aires, Argentina.

**Keywords:** Niños, animales domésticos, parasitosis intestinales, prevalencia, zoonosis, Argentina

## Abstract

El objetivo de este estudio fue examinar la prevalencia de parásitos intestinales de niños y animales domésticos en dos barrios periurbanos de la ciudad de Corrientes, Argentina. También evaluamos las características de los humanos, factores socio-ambientales y prácticas de higiene asociadas con la presencia de parásitos. Las muestras fecales fueron examinadas usando técnicas de concentración por sedimentación y flotación. Se detectaron huevos de *Enterobius vermicularis* en niños utilizando el método de Graham. El análisis de los datos fue univariado y bivariado. En total, 58 viviendas fueron evaluadas, de las cuales se obtuvieron 146 muestras de heces de niños y 101 muestras de animales. Se registró al menos una especie de parásito en 54 viviendas (93,1%). Encontramos parásitos en el 52,7% de los niños, principalmente los protozoos *Blastocystis* spp. (35,6%) y *Giardia* spp. (21,2%). Se detectaron parásitos en 67,32% de los animales, principalmente ancilostomídeos (60,7%). En conclusión, es evidente que el ambiente doméstico presenta condiciones favorables para la transmisión de estos parásitos.

## INTRODUCCIÓN

Los endoparásitos que afectan humanos y animales domésticos pueden multiplicarse en condiciones favorables y transmitirse entre los habitantes de la casa ^1^^,^^2^. Estas condiciones favorables están comúnmente asociadas a factores relacionados con el individuo (edad, sexo, estado nutricional, etc.) ^3^, y/o a factores de riesgo socioambientales como la disposición inadecuada de las heces, hábitos de higiene personal inadecuados, acceso desigual a la educación y a la salud, entre otros ^4^.

En ese sentido, el número de barrios marginales ha aumentado en Argentina, los cuales carecen de servicios básicos como agua potable, electricidad y saneamiento adecuado ^5^. Investigaciones realizadas en localidades de Argentina han reportado tasas de prevalencia general de endoparásitos en poblaciones infantiles y sus mascotas de 64,8% y 85,7% y 8,9% y 41%, respectivamente ^6^^,^^7^^,^^8^.

En particular, el 3,6% de las viviendas en la provincia de Corrientes no son adecuadas para vivir, el 6,5% carece de condiciones sanitarias básicas, el 11,0% se encuentran en hacinamiento crítico y el 37% se encuentra en zonas vulnerables (cerca de basurales o terrenos inundables) ^9^. Sin embargo, pocos estudios parasitológicos han tenido en cuenta estas variables ^10^.

Por lo tanto, consideramos que la ciudad de Corrientes, en el noreste de Argentina, podría representar un área de alto riesgo para la transmisión y mantenimiento de parásitos potencialmente zoonóticos. También identificamos importantes vacíos en la investigación en parasitología que involucra factores humanos, animales y ambientales, es decir que opere bajo el enfoque de Una Salud, lo cual dificulta la interpretación de todos estos factores en conjunto, así como la oportunidad de proponer medidas de control y prevención.

El objetivo principal de este estudio fue examinar la prevalencia de parásitos intestinales de niños y animales domésticos en dos barrios periurbanos del Departamento Capital (Corrientes, Argentina). Además, se evaluaron algunas características de los seres humanos (sexo y edad), rasgos socioambientales y prácticas de higiene como posibles factores de riesgo de infección parasitaria.

**MENSAJE CLAVE**

Motivación para realizar el estudio: Existen pocos reportes sobre parásitos intestinales en niños y animales domésticos en áreas urbanas de Argentina, cuyos hogares presentan características favorables para el mantenimiento y transmisión de parásitos de importancia zoonótica.

Principales hallazgos: Más del 50% de los niños y animales domésticos resultaron parasitados, la mayoría con patógenos zoonóticos.

Implicancias: Nuestros resultados evidenciaron la urgente necesidad de mejorar el control sanitario de niños y animales, y de implementar actividades de prevención de parasitosis intestinales en las viviendas analizadas.

## EL ESTUDIO

### Diseño del estudio

Realizamos un estudio transversal, observacional y descriptivo. El contacto con la población se llevó a cabo entre 2018 y 2021 en una escuela primaria. No se calculó el tamaño de la muestra, se incluyeron todas las unidades domésticas cuyos habitantes aceptaron participar. La unidad de análisis fue la vivienda ^2^.

### Área de estudio

El estudio se realizó en dos barrios suburbanos (A y B) del Departamento Capital de la Provincia de Corrientes (27°28’00” S, 58°50’00” O), noreste de Argentina (Figura 1). Estos barrios se caracterizan por presentar condiciones sanitarias deficientes y un alto grado de contacto con animales domésticos y sinantrópicos, entre otros.

**Figura 1:** Localización geográfica de la Provincia de Corrientes en el nordeste argentino (a), Departamento Capital (b) y los barrios periurbanos analizados (c).

### Recolección de muestras

Se entregó en cada domicilio un kit de colecta para cada niño y animal doméstico. El kit consistía de frascos colectores de heces con formaldehído al 10% y test de Graham para el diagnóstico de parásitos intestinales. Se proporcionaron explicaciones verbales a los participantes, las cuales se complementaron con instrucciones ilustradas incluidas en el kit. En el estudio participaron niños de ambos sexos con edades comprendidas entre 0 y 14 años. Se consideraron dos grupos etarios: niños no escolarizados (de 3 años o menos) y niños escolarizados (de 4 años o más). Los padres recogieron muestras de heces y mucosa perianal durante 5-6 días consecutivos. Las muestras de heces de los animales fueron recogidas por sus propietarios durante 3-4 días consecutivos o por el equipo de investigación. Se recomendó a los propietarios que tomaran las muestras de heces de los animales inmediatamente después de que estos defequen y en una zona central para evitar posibles contaminaciones.

### Recolección de datos socioambientales y demográficos

Se recogieron los datos mediante observación directa del entorno familiar y la aplicación de cuestionarios. Los datos obtenidos estaban relacionados con diferentes variables de riesgo o factores de exposición, incluidas las prácticas de higiene (Tablas 1 y 2).

**Tabla 1:** Características socio-ambientales de la población en los barrios periurbanos analizados de la ciudad de Corrientes.

Características	n (%) ^a^
Hacinamiento (personas por habitación)	
Sí	28 (50)
No	28 (50)
Presencia de animales domésticos	
Sí	52 (92,9)
No	4 (7,1)
Tipo de suelo (interior de la casa)	
Cemento u otros	49 (87,5)
Arena/tierra	7 (12,5)
Tipo de suelo (exterior de la casa)	
Cemento u otros	4 (7,1)
Arena/tierra	52 (92,9)
Fuente de agua principal	
Pozo	17 (30,3)
Red de agua comunitaria (potable)	39 (69,7)
Disposición de excretas humanas	
Letrina	34 (60,8)
Baño instalado	22 (39,2)
Riesgo de inundación	
Nunca	37 (66,1)
Ocasionalmente	19 (33,9)
Eliminación de excretas animales	
Permanece en el suelo	21 (37,5)
Desechadas en bolsas	35 (62,5)
Eliminación de aguas residuales	
Pozo ciego	55 (98,2)
Al aire libre	1 (1,8)
Eliminación de residuos sólidos	
Colecta municipal	29 (51,8)
Al aire libre o incineración	27 (48,2)
Eliminación de pañales	
Colecta municipal	23 (41,1)
Al aire libre o incineración	23 (41,1)
No utilizan pañales	10 (17,8)
Presencia de roedores	
Sí	6 (10,8)
No	46 (82,1)
Ausencia de datos	4 (7,1)
Tratamiento antiparasitario farmacológico en humanos	
Sí	15 (26,8)
No	41 (73,2)
Tratamiento antiparasitario farmacológico en animales	
Sí	3 (5,3)
No	53 (94,7)
Educación de la madre	
No escolarizada	1 (1,8)
Primaria completa	31 (55,3)
Primaria incompleta	7 (12,5)
Secundaria completa	15 (26,8)
Secundaria incompleta	1 (1,8)
Ausencia de datos	1 (1,8)
Educación del padre	
No escolarizado	1 (1,8)
Primaria completa	25 (44,6)
Primaria incompleta	6 (10,8)
Secundaria completa	9 (16,0)
Ausencia de datos	15 (26,8)

a La frecuencia se estimó en relación al total de viviendas con datos socio-ambientales (N=56).

**Tabla 2:** Prácticas de higiene de la población infantil evaluada pertenecientes a barrios periurbanos de la ciudad de Corrientes.

Prácticas de higiene	n (%)^a^
Frecuencia con la que se baña	
Más de una vez a la semana	20 (13,7)
Todos los días	126 (86,3)
Lavado de manos antes de comer y después de salir del baño	
Nunca	3 (2,0)
Ocasionalmente	52 (35,7)
Siempre	87 (59,5)
Sin datos	4 (2,8)
Mantenimiento de las uñas	
Cortas	119 (81,6)
Largas	24 (16,4)
Sin datos	3 (2,0)
Camina descalzo/a	
Nunca	28 (19,1)
Ocasionalmente	45 (30,9)
Siempre	68 (46,6)
Sin datos	5 (3,4)
Juega en la tierra	
Sí	75 (51,3)
No	67 (45,9)
Sin datos	4 (2,8)

a La frecuencia se estimó en relación al número total de niños con datos de prácticas de higiene (n=146).

### Análisis parasitológico

Todas las muestras fecales fueron examinadas en el Laboratorio de Biología de los Párasitos de la Facultad de Ciencias Exactas y Naturales y Agrimensura de la de la Universidad Nacional del Nordeste (FaCENA-UNNE) en Corrientes, Argentina, utilizando técnicas de concentración por sedimentación y flotación ^11^. Se utilizó el método de Graham para evaluar la presencia de huevos de *Enterobius vermicularis* en niños. La identificación de los elementos parasitarios (huevos, larvas, quistes, ooquistes) se basó en sus características morfológicas y medidas. Todas las familias recibieron por escrito los resultados del diagnóstico parasitológico de los niños. Asimismo, se entregó a las autoridades sanitarias correspondientes un informe técnico con los resultados obtenidos de niños y animales.

### Análisis estadístico

*Análisis univariado*

Se describieron frecuencias y porcentajes mediante análisis univariado. La prevalencia general se calculó como el número de unidades de análisis positivas y de hospedadores parasitados positivos para al menos una especie dividido por el número total de casas evaluadas y de hospedadores analizados, expresado en porcentajes; la riqueza específica se definió como el número de especies parasitarias. También se calculó el porcentaje de participantes monoparasitados y poliparasitados (dos o más especies parasitarias por hospedador).

*Análisis bivariado*

Se utilizó el Test Exacto de Fisher con el objetivo de comparar los dos barrios y evaluar la asociación entre pares de especie, así como la relación entre grupo de edad, sexo y parasitosis. El análisis de datos se llevó a cabo en el programa R (R Core Team 2022) ^12^.

Las relaciones entre el grupo de edad de los niños y las especies encontradas, así como entre el sexo y las especies se evaluaron en primer lugar mediante el Análisis de Correspondencias Múltiples. Esto permite tener una visión general de las relaciones que existen entre las variables para luego verificar tales asociaciones (mediante una prueba de Chi-cuadrado o la prueba exacta de Fisher). Se utilizó el mismo método para analizar la relación de las características socioambientales con las parasitosis y las especies parasitarias y la relación de las prácticas de higiene con las parasitosis y las especies parasitarias. No realizamos análisis multivariado.

### Aspectos éticos

El estudio fue aprobado por la Secretaría de Ciencia y Tecnología de la Universidad Nacional del Nordeste (UNNE), Argentina. Esta investigación también se llevó a cabo de conformidad con las leyes argentinas. La recolección de muestras se realizó bajo permisos oficiales de acuerdo con la Declaración Universal de los Derechos Humanos de 1948, las normas éticas establecidas por el Código de Nuremberg de 1947, la Declaración de Helsinki de 1964 y sucesivas modificaciones, así como las disposiciones de la Ley Nacional 25.326 de protección de datos personales.

## HALLAZGOS

### Viviendas

Se registró un total de 65 hogares con 164 niños y 205 animales domésticos. Al menos 58 hogares (considerados para el análisis) proporcionaron una muestra de heces humanas y/o animales. Se recogieron 146 muestras de niños y 101 de animales, lo que representa un porcentaje de retorno del 89,0% y el 49,2%, respectivamente.

Se registró la presencia de al menos una especie de parásito y/u organismo no patógeno de niños y/o animales hospedadores en 54 viviendas (93,1%). La riqueza específica fue de 17 especies, con un máximo de siete especies en una sola vivienda. Los protozoos más prevalentes fueron *Blastocystis* spp. y *Giardia* spp., en el 60,3% y el 43,1% de las viviendas, respectivamente, mientras que los helmintos más prevalentes fueron ancylostoma (46,5%) y *Enterobius vermicularis* (15,5%). Se registró una mayor prevalencia general en el barrio A (n=30) que en el barrio B (n=28) (96,6% frente a 89,2%), pero esta diferencia no fue estadísticamente significativa (p > 0,05).

### Análisis socioambiental

Se obtuvieron datos socioambientales de 56 viviendas, además de datos sobre las prácticas de higiene personal de 146 niños (Tablas 1 y 2). Encontramos asociaciones estadísticas entre la eliminación de residuos sólidos y la presencia *Hymenolepis* nana (p=0,024), así como entre la frecuencia con la que se bañan los participantes y la presencia de *Entamoeba coli* (p=0,042)(Tabla 3).

**Tabla 3:** Variables seleccionadas por asociación estadísticamente significativa ^a^.

Variables	Parasitosis	Especies parásitas ^b^	
		*Hymenolepis nana*	*Entamoeba coli*
Grupo de edad			
Escolarizados	p=0,048	-	-
No escolarizados	Referencia	-	-
Eliminación de residuos sólidos			
Colecta municipal	-	p=0,024	-
Al aire libre o incineración	-	Referencia	-
Frecuencia con la que se baña			
Más de una vez a la semana	-	-	Referencia
Todos los días	-	-	p=0,042

a Se utilizó la prueba exacta de Fisher para evaluar la independencia de las variables.

b Se evaluaron las demás especies parásitas, pero solo se muestran aquellas con asociación significativa con las variables.

### Análisis parasitológico en niños

Se analizaron 72 niñas y 74 niños de 0 a 14 años (45 no escolarizados y 101 escolarizados). Se encontró al menos una especie de parásito en 77 muestras (52,7%). La riqueza específica fue de seis especies, siendo el protozoo *Blastocystis* spp. (35,6%) la especie más prevalente, seguido de *Giardia* spp. (21,2%). Encontramos mayor presencia de parásitos en niños en edad escolar (4 años y más) que en aquellos que no se encontraban en edad escolar (3 años y menos) (40,4% y 12,3% respectivamente; p< 0,05) (Tabla 4).

La mayoría de muestras presentaron una sola especie de parásito, mientras que las muestras con múltiples parásitos presentaron un máximo de cuatro especies. Se observó una asociación estadísticamente significativa entre la presencia de *Giardia* spp. y *E. vermicularis* (x^2^=7,3; p< 0,05).

### Análisis parasitológico en animales

Se analizó un total de 101 muestras fecales de animales domésticos; 68 (67,32%) presentaron al menos una especie de parásito. La riqueza específica fue de 15 especies. La mayoría de los animales analizados presentaron una sola especie de parásito (Tabla 4).

**Tabla 4:** Prevalencia general y de especies parásitas en niños (N=146) y animales (N=101) de dos barrios periurbanos del Departamento Capital, Provincia de Corrientes.

Grupos o especies parásitas	Número de muestras positivas en niños n (%)	Grupo de edad Número de muestras positivas n (%)		Número de muestras positivas en animales n (%)				
		Niños no escolarizados	Niños escolarizados	Perros (n=84)	Caballos (n=8)	Cerdos (n=5)	Gatos (n=2)	Conejos (n=2)
General	77 (52,7)	18 (12,3)	59 (40,4)	60 (71,4)	2 (25,0)	4 (80,0)	1 (50,0)	1 (50,0)
Poliparasitados	28 (36,3)	6 (33,3)	23 (38,9)	16 (26,6)	0	1 (25,0)	1 (100)	0
Monoparasitados	49 (63,6)	12 (66,6)	36 (61,0)	44 (73,3)	2 (100)	3 (75,0)	0	1 (100)
Párasitos								
Protozoos	70 (47,9)	17 (11,6)	55 (37,6)	18 (21,4)	2 (25,0)	3 (60,0)	1 (50,0)	1 (50,0)
*Blastocystis* spp.	52 (35,6)	13 (8,9)	39 (26,7)	2 (3,5)	0	0	0	1 (50,0)
*Coccidia*	0	0	0	2 (2,3)	2 (25,0)	1 (20,0)	0	0
*Eimeria* spp.	0	0	0	1 (1,1)	0	0	0	0
*Entamoeba coli*	10 (6,8)	2 (1,3)	8 (5,4)	1 (1,1)	0	1 (20,0)	0	0
*Giardia* spp.	31 (21,2)	9 (6,1)	22 (15,0)	11 (13,0)	0	0	0	0
*Iodamoeba bustschlii*	0	0	0	0	0	1 (20,0)	0	0
*Isospora canis*	0	0	0	1 (1,1)	0	0	1 (50,0)	0
Helmintos	24 (16,4)	4 (2,7)	18 (12,3)	53 (63,0)	0	2 (40,0)	1 (50,0)	0
*Ascaris lumbricoides*	3 (2,0)	1 (0,6)	2 (1,3)	0	0	0	0	0
*Dipylidium caninum*	0	0	0	2 (2,3)	0	0	0	0
*Enterobius vermicularis*	15 (10,2)	2 (1,3)	12 (8,2)	0	0	0	0	0
Ancylostoma	0	0	0	51 (60,7)	0	0	1 (50,0)	0
*Hymenolepis nana*	5 (3,4)	1 (1,3)	4 (2,7)	1 (1,1)	0	0	0	0
*Oesophagostomum* spp.	0	0	0	0	0	2 (40,0)	0	0
*Spirometra* spp.	0	0	0	2 (2,3)	0	0	0	0
Larva de *Strongyloides*	0	0	0	1 (1,1)	0	0	0	0
*Toxocara canis*	0	0	0	7 (8,3)	0	0	1 (50,0)	0
*Trichiuris vulpis*	0	0	0	4 (4,7)	0	0	0	0

## DISCUSIÓN

Los animales infectados eliminan estadios parasitarios a través de sus heces y contaminan el ambiente ^13^, en este sentido, nuestros resultados muestran que el 58,6% de las viviendas estaban contaminadas con especies parásitas (particularmente geohelmintos) encontradas en heces de animales, la mayoría con potencial zoonótico. El 39,6% de los hogares tenían un ambiente contaminado además de la presencia de especies parásitas en heces de niños y el 32,7% de las viviendas registraban sólo la presencia de especies parásitas en heces de niños (principalmente protozoos zoonóticos). Por lo tanto, es evidente que en estas viviendas las condiciones para el desarrollo del ciclo vital, el mantenimiento y la transmisión tanto de helmintos como de protozoos son óptimas y constituyen un escenario epidemiológico grave para la salud pública.

Se encontraron parásitos en más del 50% de los niños, la mayoría de ellos presentaron una sola especie. Esto coincide con investigaciones previas realizadas en Corrientes ^10^^,^^14^ y otras zonas de Argentina ^2^^,^^15^. Las especies de protozoos más prevalentes fueron *Blastocystis* y *Giardia* (35,6% y 21,2%, respectivamente), mientras que la prevalencia de *Entamoeba coli* (6,8%) fue la menor. Este patrón ha sido reportado en poblaciones infantiles de Argentina ^7^ y de la provincia de Corrientes, con prevalencia similar a la descrita en nuestros resultados ^10^; sin embargo, más recientemente, a nivel local se encontró que la prevalencia de *Blastocystis* era considerablemente menor (16,7%) ^14^.

*Giardia* es conocida a nivel mundial como uno de los principales parásitos causantes de enfermedades diarreicas (no víricas ni bacterianas) en humanos y otros mamíferos ^16^; mientras que *Blastocystis* se ha asociado a diferentes enfermedades intestinales y extraintestinales en humanos ^17^. *Entamoeba coli* es comensal, pero es un indicador de contaminación fecal ambiental. Estos tres protozoos comparten la misma vía de transmisión fecal-oral y su presencia está asociada al agua contaminada o al consumo de vegetales crudos con heces de huéspedes infectados ^18^. También se han descrito deficiencias en los hábitos de higiene personal, particularmente en el lavado de manos ^19^. En ese sentido, se ha reportado que los padres tienden a informar excesivamente sobre las conductas de lavado de manos de sus hijos, lo que disminuiría sistemáticamente cualquier beneficio aparente y en algunos casos incluso podría mostrar efectos negativos ^20^. En este sentido, aunque las madres declararon que la mayoría de los niños se lavaban las manos antes de comer y después de salir del baño, observamos deficiencias en la higiene general de los niños, situación también reportada por las autoridades escolares. Por lo tanto, este tipo de resultado debe ser validado con una metodología que permita la observación directa. Sorprendentemente, la presencia de *E. coli* se asoció con la frecuencia con la que se bañan los individuos, pero el 86,3% de las madres declararon que sus hijos se bañaban todos los días, lo que apoya la necesidad de la observación directa.

En cuanto a los animales, se registraron *Giardia* y *Blastocystis* en muestras de heces de perros (13,0% y 3,5%, respectivamente) y en un conejo. Se encontró *Entamoeba coli* en un perro y un cerdo. En la ciudad de La Plata, Cociancic *et al*. ^2^ reportaron una tasa de prevalencia similar en perros para *Giardia* y una prevalencia mayor para *E. coli* (10,3%), pero no encontraron *Blastocystis*. La prevalencia fue aún menor para estos protozoos en perros de otra localidad de Argentina ^15^. En definitiva, a pesar de la baja prevalencia de estos parásitos en mascotas, establecemos el papel de los animales como fuente de contaminación ambiental y diseminadores o como posibles focos de transmisión animal-animal o humano-animal, en este caso son necesarios estudios moleculares para comprobar posibles transmisiones cruzadas.

En cuanto a los helmintos, registramos principalmente *Enterobius vermicularis* (10,2%) y, en menor medida, *Hymenolepis nana* (3,4%) y *Ascaris lumbricoides* (2,0%). *Enterobius vermicularis* ha sido ampliamente reportado en todas las provincias de Argentina, con una prevalencia que oscila entre el 13,6% y el 50,9% ^14^. Diferentes estudios asociaron su presencia con la onicofagia ^2^, el deficiente lavado de uñas y manos, el hacinamiento y el compartir camas y ropa ^21^. En nuestro estudio, es posible que la baja prevalencia esté relacionada con una ineficiente toma de muestra por parte de los padres.

*Hymenolepis nana* comúnmente infecta a humanos y roedores. En Argentina se ha reportado una prevalencia similar a la encontrada en nuestro trabajo ^14^^,^^22^. En contraste, en la provincia de Misiones se reportó una tasa de prevalencia mayor al 20,0% ^7^. Nuestros resultados muestran que la presencia de este parásito se asoció con la eliminación de residuos sólidos. Sin embargo, en todos los hogares donde se encontró este helminto, los adultos declararon que la basura es recogida en bolsas y luego retirada por el servicio de recolección municipal. Es importante señalar que observamos acumulación excesiva de basura tanto a nivel de viviendas como en sectores específicos de los barrios evaluados. Por lo tanto, se evidencia una inadecuada disposición de residuos en esta comunidad, y consideramos que esto representa un riesgo, dada la presencia no solo de roedores sino también de animales domésticos que se estuvieron en contacto directo con los residuos.

Se encontró *A. lumbricoides* en tres niños de tres viviendas diferentes. En cambio, en otras zonas de Argentina se ha reportado una alta prevalencia de este parásito (38,9%) ^23^. Su presencia en el medio ambiente está relacionada principalmente con la defecación al aire libre, las condiciones climáticas y el suelo, estos últimos, determinantes de la viabilidad y maduración de los huevos. En Brasil, Gonçalves *et al.*^24^ determinaron la ausencia de letrinas como un factor de riesgo, mostrando que en América del Sur los pueblos indígenas suelen vivir en condiciones de extrema pobreza.

Con respecto a animales, los ancylostomas, *Toxocara canis* y *Trichuris vulpis* se reportaron con mayor prevalencia en perros (60,7%, 8,3% y 4,7%, respectivamente). Esto demuestra una gran contaminación fecal canina y una deficiente atención sanitaria de las mascotas en las viviendas. Resultados similares fueron reportados en la ciudad de Corrientes por Milano *et al*. ^1^, y valores más altos fueron reportados en la ciudad de La Plata por Cociancic *et al*. ^3^. Las larvas de *ancylostoma* y *T. canis* pueden transmitirse a los seres humanos y causar el síndrome de larva migratoria cutánea y el síndrome de larva migrans neural, respectivamente ^25^. *Trichuris vulpis* es un nematodo del intestino grueso de los perros y tiene importancia en la medicina veterinaria.

Por otra parte, en coincidencia con Rivero *et al.*^15^ la coinfección de *Giardia* spp. y *E. vermicularis* resultó la más común y estadísticamente siginificativa, esto podría deberse a la vía feco-oral de transmisión que ambas especies parásitas comparten.

En cuanto a las variables demográficas, encontramos una asociación significativa entre el grupo de edad y la parasitosis, siendo los escolares el grupo con mayor prevalencia de parásitos. Resultados similares han sido reportados por Rivero *et al*. ^15^ y Navone *et al*. ^14^. Este hallazgo podría atribuirse a que los escolares mantienen un estrecho contacto con los focos de infección parasitaria a través del juego y hábitos de higiene insuficientes ^26^.

El tamaño de muestra fue una de las limitaciones de nuestro estudio, debido a que trabajamos con un bajo número de viviendas y un bajo número de heces de animales y humanos.

En conclusión, encontramos que gran parte de las viviendas estaban contaminadas con especies parásitas, en su mayoría helmintos y protozoos de importancia zoonótica, lo cual evidencia la urgente necesidad de mejorar el control sanitario de niños y animales en el área analizada.
